# Spinosad and the Tomato Borer *Tuta absoluta*: A Bioinsecticide, an Invasive Pest Threat, and High Insecticide Resistance

**DOI:** 10.1371/journal.pone.0103235

**Published:** 2014-08-14

**Authors:** Mateus R. Campos, Agna Rita S. Rodrigues, Wellington M. Silva, Tadeu Barbosa M. Silva, Vitória Regina F. Silva, Raul Narciso C. Guedes, Herbert Alvaro A. Siqueira

**Affiliations:** 1 Departamento de Agronomia (Entomologia), Universidade Federal Rural de Pernambuco, Recife, PE, Brazil; 2 Departamento de Entomologia, Universidade Federal de Viçosa, Viçosa, MG, Brazil; Institute of Vegetables and Flowers, Chinese Academy of Agricultural Science, China

## Abstract

The introduction of an agricultural pest species into a new environment is a potential threat to agroecosystems of the invaded area. The phytosanitary concern is even greater if the introduced pest’s phenotype expresses traits that will impair the management of that species. The invasive tomato borer, *Tuta absoluta* (Meyrick) (Lepidoptera: Gelechiidae), is one such species and the characterization of the insecticide resistance prevailing in the area of origin is important to guide management efforts in new areas of introduction. The spinosad is one the main insecticides currently used in Brazil for control of the tomato borer; Brazil is the likely source of the introduction of the tomato borer into Europe. For this reason, spinosad resistance in Brazilian populations of this species was characterized. Spinosad resistance has been reported in Brazilian field populations of this pest species, and one resistant population that was used in this study was subjected to an additional seven generations of selection for spinosad resistance reaching levels over 180,000-fold. Inheritance studies indicated that spinosad resistance is monogenic, incompletely recessive and autosomal with high heritability (*h* ^2^ = 0.71). Spinosad resistance was unstable without selection pressure with a negative rate of change in the resistance level ( = −0.51) indicating an associated adaptive cost. Esterases and cytochrome P450-dependent monooxygenases titration decreased with spinosad selection, indicating that these detoxification enzymes are not the underlying resistance mechanism. Furthermore, the cross-resistance spectrum was restricted to the insecticide spinetoram, another spinosyn, suggesting that altered target site may be the mechanism involved. Therefore, the suspension of spinosyn use against the tomato borer would be a useful component in spinosad resistance management for this species. Spinosad use against this species in introduced areas should be carefully monitored to prevent rapid selection of high levels of resistance and the potential for its spread to new areas.

## Introduction

Invasive agricultural pest species are widely recognized as a major threat to agroecosystems and agricultural production [1]–[3]. An additional phytosanitary concern is that the introduced pest’s phenotype could include inheritable traits that could impose management difficulties, such as resistance to insecticides [4]–[7]. The invasive species, *Tuta absoluta* (Meyrick) (Lepidoptera: Gelechiidae), the tomato borer or tomato leafminer (also tomato pinworm), is one such species. It is of South American origin but was introduced into Europe as early as 2006. This pest has subsequently spread to North Africa and the Middle East and is now threatening the whole of Asia, particularly China and India, the two leading world tomato producers [8]–[11].

From its Peruvian origin, the tomato borer has spread in South America. Its eventual introduction into Brazil, the leading neotropical tomato producer [11], led to drastic changes in tomato production in the country with a dramatic increase in insecticide use in the early 1980’s [10]. Problems with insecticide resistance in the tomato borer were soon detected in the late 1990’s and early 2000’s in Chile, Brazil and Argentina for the insecticides initially used against this species, including organophosphates, pyrethroids, abamectin and cartap [12]–[17]. This resistance led to subsequent registration and large-scale use of new insecticides, particularly in Brazil, including insect growth regulators, indoxacarb, chlorfenapyr, spinosyns, and diamides [10], [18], [19]. Organically produced tomatoes imposed additional restrictions and challenges for tomato borer control, culminating in the use of bioinsecticides, such as the spinosyn spinosad and *Bacillus thuringiensis* Berliner, aided by alternative supporting control methods [10], [18].

Insecticide registration and use against the tomato borer in South America led to corresponding waves of change in the prevailing patterns of insecticide resistance congruent with the patterns of insecticide use and control failures [13], [19], [20], [21]. The trends, closely followed in Brazil, were intensive use of chitin synthesis inhibitors succeeded the use of abamectin, cartap and pyrethroids against the borer in tomato fields, reaching high levels of resistance (>100-fold), followed by evidences of control failure with this group of insect growth regulators [20], [21]. The bioinsecticide spinosad, a compound of natural origin used in neotropical tomato fields (both organic and conventional fields), has become one of the main compounds used against the tomato borer, but reports of resistance have started to appear both in Brazil and Chile [21], [22], [23].

The appeal of spinosad, a fermentation product of the soil actinomycete *Saccharopolyspora spinosa* (Mertz and Yao), includes its safety profile and acceptable use in organically produced tomatoes [24]–[26]. However, spinosad resistance counterpoints this appeal and the potential for further use. More seriously, the swift development of insecticide resistance in neotropical field populations of the tomato borer is suggestive of a rapid evolution of spinosad resistance, which remains to be tested [19], [21]. The introduction of the tomato borer from South America into Europe, suggests additional problems for managing this destructive species in newly infested areas, further threatening the current world tomato production [19].

The high genetic homogeneity reported among populations of the tomato borer from South America and Europe give credence to the apparent high level of dispersion of the species and a shared origin [27], [28]. These findings also support the hypothesis of a single invasive event for the tomato borer in Europe [19], [28]. The emerging studies of insecticide resistance in Europe and, particularly, the survey of pyrethroid resistance due to altered target site sensitivity also provides support for the single-introduction event of the tomato borer [29]–[31]. Additionally, the introduced borer phenotype was likely resistant to at least pyrethroid insecticides [19], [31], but may also be capable of rapid development of resistance to other insecticides, including bioinsecticides widely used in traditional and organic tomato production.

In our study, a field population of the tomato borer, already exhibiting spinosad resistance, was subjected to further selection for spinosad resistance to assess the rate of development and level of resistance likely to be achieved with intensive use of this insecticide. The spinosad-selected strain of tomato borer was also subsequently used for the genetic characterization of spinosad resistance and assessment of its stability. This strain was also utilized to evaluate the potential involvement of detoxification by esterases and cytochrome P450-dependent monooxygenases as the underlying resistance mechanism and to assess its cross-resistance spectrum. Based on previous findings in Brazil, a fast response to spinosad selection, reaching high levels of resistance (>100-fold) in few generations (<10) and monogenic resistance was expected. The involvement of cytochrome P450-dependent monooxygenases was previously suggested in Chilean populations of the tomato borer [22]. Evidence of cross-resistance has not yet been detected in the tomato borer, except within pyrethroids and chitin synthesis inhibitors [13], [20], [31]. Therefore, cross-resistance is more likely among spinosyns than between spinosad and insecticides from other groups, especially if altered target site sensitivity is involved.

## Materials and Methods

### Ethics Statement

This study did not involve any endangered or protected species. Although the insect species studied is a pest species, permits were secured for the collection of the original field populations. The laboratory colonies were initially established from over 200 field-collected individuals.

### Insects

Populations of the tomato borer were collected from experimental and commercial tomato fields during 2010/2011 in four regions in Brazil. These insect populations were subjected to an initial screening for spinosad resistance and the populations from Iraquara (state of Bahia, Brazil) and Pelotas (state of Rio Grande do Sul, Brazil) were used for our experiments. The insects were laboratory-maintained in wooden cages with anti-aphid mesh. The cages were separate in larvae cage (45×45×45 cm) and adult cage (30×30×30 cm). The adult cage was used for oviposition only, where leaves of tomato were provided daily as substrate. Adults of *T. absoluta* were fed with 10% glucose solution (Yoki, 10 Brazil), while the larvae were fed with tomato leaves from Santa Clara tomato cultivar (IC11 5500), cultivated under greenhouse conditions without any insecticide application [20], [21]. The insects were maintained under the controlled conditions of 25±1°C temperature, 65±5% relative humidity and 12∶12 (L:D) photoperiod.

### Insecticides

The bioinsecticide spinosad was used in its commercial formulation registered for use in tomato fields against the tomato borer (480 g a.i./L, suspension concentrate, Dow AgroSciences, Franco da Rocha, SP, Brazil) [18]. The insecticides used in the cross-resistance bioassays were (the commercial formulations used are indicated between parentheses): abamectin (18 g a.i./L, emulsifiable concentrate, Syngenta Proteção de Cultivos, São Paulo, SP, Brazil), cartap (500 g a.i./Kg, soluble powder, Iharabras, Paulínia, SP, Brazil), chlorantraniliprole (200 g a.i./L, suspension concentrate, DuPont Brazil, Paulínia, SP, Brazil), chlorfenapyr (240 g a.i./L, suspension concentrate, BASF S.A., São Paulo, SP, Brazil), chlorpyrifos (480 g a.i., emulsifiable concentrate, Dow AgroSciences, Santo Amaro, SP, Brazil), indoxacarb (300 g a.i./Kg, water dispersible granule, DuPont Brazil, Paulínia, SP, Brazil), permethrin (384 g a.i./L, emulsifiable concentrate, FMC Química do Brazil, Campinas, SP, Brazil), spinetoram (250 g a.i./Kg, water dispersible granule, Dow AgroSciences, Franco da Rocha, SP, Brazil), and thiamethoxam (250 g a.i./Kg, water dispersible granule, Syngenta Proteção de Cultivos, São Paulo, SP, Brazil). The synergists piperonyl butoxide (PBO-90%) and *S,S,S* – Tributylphosphorotrithioate (DEF-98%) were purchased from Sigma-Aldrich, Milwaukee, WI, EUA).

### Concentration-mortality bioassays

The concentration-mortality bioassays were performed as described previously and validated for the tomato borer, *T. absoluta* [21]–[23], [32]. The insecticide solutions were diluted in water containing 0.01% Triton X-100 and a control treatment without insecticide was used to record natural mortality. Insecticide-treated tomato leaves were placed in Petri dishes (9 cm diameter) with ten 2^nd^ instar larvae of the tomato borer and were maintained under controlled environmental conditions (25±1°C temperature, 65±5% relative humidity and 12∶12 (L:D) photoperiod). Larval mortality was assessed after 48 hours of exposure by prodding the insects with a fine hairbrush. Larvae were considered dead if they were unable to move the length of their body.

### Selection for spinosad resistance

The tomato borer population from Iraquara, previously identified as resistant to spinosad [23], was subjected to spinosad selection after four generations under laboratory conditions. The original Iraquara population was split into two lines, one maintained without insecticide exposure and the other maintained under spinosad selection for 22 generations. Between 1,500 and 2,000 2^nd^ instar larvae of the tomato borer surviving exposure to increasing discriminatory concentrations of spinosad (selected based on the concentration-mortality bioassays) were used for selection in each generation. After the reduction in egg laying by the spinosad-selected population following the 8^th^ generation of selection, the discriminating concentration of 500 µg a.i./mL was maintained. The average mortality of the spinosad-selected population of Iraquara between the F_2_ and F_7_ generations was used to estimate the heritability of spinosad resistance. The Pelotas population of the tomato borer, previously identified as susceptible to spinosad, was maintained in the laboratory without insecticide selection as a susceptible standard population.

### Stability of spinosad resistance

The spinosad-resistant population selected for 13 generations was split into two lines, one was maintained under spinosad selection as previously described, and the other was maintained without spinosad selection. Both lines were subjected to spinosad concentration-mortality bioassays during each subsequent generation until the 22^nd^ generation to verify the stability of spinosad resistance without the selection pressure of the bioinsecticide.

### Inheritance of spinosad resistance

The inheritance of spinosad resistance was determined through reciprocal crosses between spinosad-selected insects (after 13 generations of selection) and susceptible insects (from Pelotas). Thirty-five crosses were performed for each reciprocal cross with the adults maintained in separate rearing cages for progeny production and concentration-mortality bioassays. The LC_50_ values (and the LC_90_ values) were estimated for both parental strains and reciprocal crosses were used to calculate the degree of dominance (D) of spinosad resistance [38]–[40]. The estimated dominance (*h*) of spinosad resistance was tested through concentration-mortality bioassays with spinosad for the parental (susceptible and (selected) spinosad-resistant) strains and the F_1_ progeny from the reciprocal crosses [38]. Five spinosad concentrations (0.005, 0.05, 0.5, 5 and 10 µg a.i./mL), in addition to untreated controls (with the application of only water and adjuvant), were used against individuals of the pooled F_1_ of reciprocal crosses (n = 180), spinosad-resistant (n = 135), and spinosad-susceptible (n = 177) populations.

The monogenic basis of spinosad resistance was tested using backcrosses with the F_1_ individuals obtained in the reciprocal crosses between spinosad-selected parental insects. The 2^nd^ instar larvae obtained from such backcrosses were subjected to concentration-mortality bioassays with spinosad and to a direct test of inheritance to recognize their mono or polygenic basis.

### Pattern of cross-resistance

The 2^nd^ instar larvae of the 15^th^ and 16^th^ generations of spinosad selection were used in concentration-mortality bioassays with the insecticides abamectin, cartap, chlorantraniliprole, chlorfenapyr, chlorpyrifos, indoxacarb, permethrin, spinetoram and thiamethoxam to detect the potential spectrum of spinosad cross resistance. The bioassay methods used were those previously described for the concentration-mortality bioassays.

### Synergism of spinosad

The 2^nd^ instar larvae of spinosad susceptible and resistant colonies were used in concentration-mortality bioassays with the insecticides spinosad + PBO and spinosad + DEF to detect whether metabolism is involved in the resistance. The bioassay methods used were those previously described for the concentration-mortality bioassays, but all larvae were topically treated (0.2 µL/larvae) with a concentration of either PBO (1 µg/µL) or DEF (1 µg/µL) before exposure to spinosad.

### Protein extraction and enzyme bioassays

Three batches of ten 3^rd^ instar larvae were collected during each generation of the spinosad selection for triplicate determinations of enzyme activity. The crude insect homogenate was prepared by grinding ten larvae in 0.2 mL of sodium phosphate buffer (0.02 M, pH 7.2). The crude homogenate was filtered through glass-wool and centrifuged at 10,000 *g_max_* for 15 min. The pellet was discarded and the supernatant was used for determining protein content and total esterase activity. The supernatant from the 10,000 *g_max_* centrifugation was further centrifuged at 100,000 *g_max_* to obtain the microsomal fraction, which was resuspended in 500 µL sodium phosphate [0.1 M, pH 7.5+ glycerol (20%)] and used for the determination of cytochrome P450 *O-*demethylase activity.

Protein concentration was determined following the bicinchoninic acid method using bovine serum albumin as standard [33]. Total esterase activity was determined following the methods of van Asperen [34] using α-naphthyl acetate as substrate and a standard curve of α-naphthol to estimate the esterase activity expressed in nmol α-naphthol/min/mg protein. Cytochrome P450 (*O*-demethylase) activity was determined using *p*-nitroanisole as substrate generating *p*-nitrophenol [35] and enzyme activity was expressed in nmol *p*-nitrophenol/min/mg protein.

### Statistical analyses

The concentration-mortality data were subjected to probit analysis using the software Polo-Plus (LeOra Software Co., Petaluma, CA, USA) with correction for the natural mortality (without insecticide exposure) in the bioassays [36]. The level of resistance was estimated using resistance ratio (RR) estimates, which were considered significant when the 95% confidence interval of the RR did not include the value 1.0 [37].

The stability of spinosad resistance was estimated based on the average response of the spinosad-selected population between the 13^th^ and 18^th^ generations, corresponding to the average rate of change in the absence of the insecticide (RC). Spinosad resistance is unstable if the rate of change in the absence of spinosad is negative (RC<0). The number of generations required for a 10-fold reduction in spinosad resistance (G) can also be used to estimate the average rate of change in resistance without insecticide exposure using the formula G = RC^−1^ [38], [39].

The degree of dominance (D) of spinosad resistance was calculated according to the method of Hartl [38] and Stone [40], using the formula D = 2 (2.*L_2_−L_1_−L_3_*)/(*L_1_–L_3_*), where *L_1_, L_2_*, and *L_3_* are the log values of the LC_50_s (or of LC_90_s) of the spinosad-selected, F_1_ (between resistant and selected strains), and spinosad-susceptible strains, respectively. The values of D may range from −1 to +1, with the former corresponding to complete recessive inheritance and the later to complete dominance [40].

The estimated dominance (*h*) was calculated for each concentration: *h* = (*w*12–*w*22)/(*w*11–*w*22); where *w*11, *w*12, and *w*22 represent fitness values determined for resistant homozygotes, heterozygotes, and susceptible homozygotes, respectively [38]. The fitness value of resistant homozygotes was considered 1.0, while the fitness values of heterozygotes and susceptible homozygotes were calculated as the ratio between the observed survival rate of the pooled F_1_ progeny of the reciprocal crosses and the survival rate of the (selected) spinosad-resistant strain. The *h*-values varies from 0 (completely recessive) to 1 (completely dominant), where 0.5 corresponds to co-dominance, 0<*h*<0.5 corresponds to incompletely recessive and 0.5<*h*<1.0 corresponds to incompletely dominant.

The monogenic or polygenic basis of spinosad resistance in the tomato borer was initially estimated by comparing the slopes of the concentration-mortality curves of the F_1_ reciprocal crosses (between resistant and susceptible lines) and their backcrosses. The results from the backcrosses were compared with the monogenic expectation using the χ^2^ test [41], [42]. The minimal number of effective genes (n*_E_*) was estimated with the formula n*_E_* = (*L*_2_ *–L_1_*)^2^/8σ*_s_* ^2^ [43], where *L_2_* and *L_1_* are the log of LC_50_ for the spinosad-selected strain and for the susceptible strain, respectively. The phenotypic variance (σ_s_^2^) was estimated with the formula σ*_s_* ^2^ = σ*_B1_* ^2^+σ*_B2_* ^2^–(σ*_F1_* ^2^+½.σ*_P1_* ^2^+½.σ*_P2_* ^2^), where σ*_F1_* ^2^, σ*_B1_* ^2^, σ*_P1_* ^2^ and σ*_P2_* ^2^ are the phenotypic variances of the F_1_ progeny, of the F_1_-spinosad resistance backcross progeny, of the spinosad resistant strains, and of the spinosad susceptible strain, respectively. The F_1_-spinosad susceptible backcross was not carried out and, therefore, we considered σ*_B2_* ^2^ = 0.

The heritability (*h^2^*) of spinosad resistance was estimated using the formula *h^2^* = R/S, where R is the response to selection and S is the differential selection; the 10-fold increase in spinosad resistance was estimated using the formula G = R^−1^ [38], [39]. The response to selection (R) was calculated by the formula R = (*L*_f_–*L_i_*)/*n*, where *L_f_* and *L_i_* are the log LC_50_ of the 2^nd^ and 7^th^ generations and *n* is the number of generations under selection. The differential selection (S) was estimated with the formula S = *i*.σ_F_, where *i* is the selection intensity and σ_F_ is the phenotypic standard deviation [39]. The selection intensity (*i*) was estimated for *p*, which is the percentage of individuals surviving the selection [40]. The phenotypic standard deviation (σ_F_) was estimated using the formula σ_F_ = ½.(β*_i_*+β_f_)^−1^, where β*_i_* is the initial slope and β_f_ is the final slope of the concentration-mortality curve.

The synergism ratio was calculated dividing the LC_50_ unsynergized by the LC_50_ synergized for each colony and synergist. The results of enzyme activity were subjected to analyses of variance and Tukey’s HSD test (*P*<0.05) when appropriate, after ascertaining normality and homoscedasticity assumptions (PROC GLM and PROC UNIVARIATE) [44]. Linear regression analyses between enzyme activity and LC_50_s at each generation of spinosad selection were performed using the procedure PROC REG in SAS [44].

## Results

### Selection for spinosad resistance

**Figure 1:**
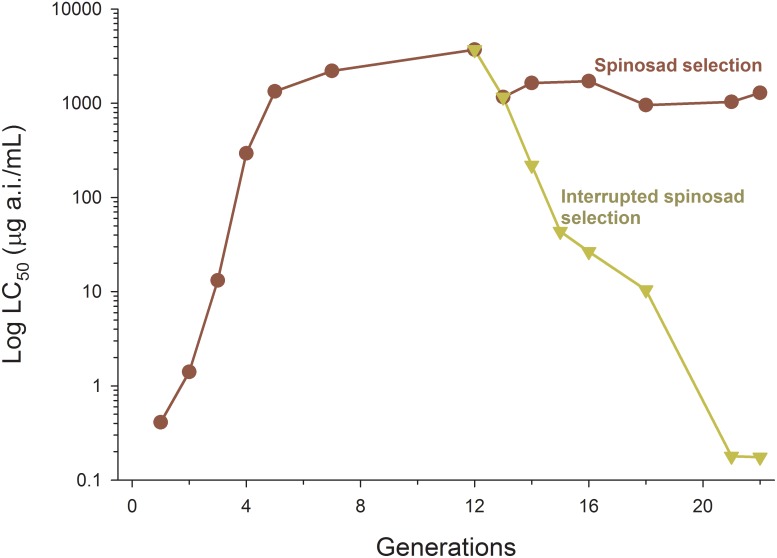
LC_50_s for spinosad with successive selections for spinosad resistance of the tomato borer *Tuta absoluta*. After 12 generations of spinosad selections, the selected line was split into two, one line maintaining selection and one line with interrupted selection.

The selection for spinosad resistance at each generation of the initial Iraquara population led to a steady increase of the level of spinosad resistance until the 7^th^ generation of selection, reaching a 5,000-fold increase in the level of resistance (>180,000-fold based on standard susceptible stain) (Table 1, Fig. 1). The selection response (R) was 0.53 and the differential selection (S) was estimated at 0.75, leading to a high heritability of spinosad resistance (*h^2^* = 0.71) and representing a 10-fold increase in the level of resistance at each 1.88 generations (Table 2).

**Table 1:** Relative toxicity of spinosad to successive generations of spinosad–selected and –unselected strains of the tomato borer *Tuta absoluta*.

Population	Generation	Degrees of freedom	Slope ± SE	LC_50_ (95% CI) (µg a.i./mL)	LC_99_ (95% CI) (µg a.i./mL)	RR_50_ (95% CI)	?^2^
Unselected	F1	5	1.85±0.23	0.41 (0.30–0.52)	7.37 (4.36–17.04)	–	1.51
Spinosad-selected	F2	6	1.21±0.18	1.41 (0.882–2.14)	117.77 (42.813–703.52)	3.43 (3.25–3.61)	1.51
	F3	6	1.06±0.20	13.13 (4.08–43.74)	2048.90 (270.67–0.28×10^7^)	32.01 (32.44–32.58)	9.91
	F4	5	1.14±0.26	294.15 (147.85–520.62)	3166.00 (7419.20–0.13×10^7^)	717.14 (716.26–718.01)	2.66
	F5	5	1.98±0.31	1333.67 (718.99–2109.73)	19760.00 (9020.30–0.11×10^6^)	3256.03 (3255.62–3258.03)	5.24
	F7	6	1.16±0.18	2200.41 (1051.24–3944.73)	22.09×10^7^ (6.0×10^7^–3.9×10^9^)	5380.88 (5379.52–5382.24)	6.74
	F12	5	1.70±0.36	3706.34 (2055.99–6152.69)	86640.00 (32808.00–0.82×10^6^)	9030.57 (9028.39–9032.74)	1.98
	F13	4	1.45±0.27	1180.48 (698.73–1756.64)	47952.00 (18525.00–0.35×10^6^)	2880.06 (2878.68–2881.44)	0.79
	F14	4	1.62±0.20	1637.63 (1240.59–2117.19)	44902.00 (23312.00–0.13×10^6^)	3993.27 (3993.08–3993.37)	3.16
	F16	4	1.47±0.19	1717.33 (998.16–2764.20)	65180.00 (21576.00–0.90×10^6^)	4191.55 (4192.49–4192.61)	5.46
	F18	5	1.08±0.19	956.66 (542.62–1537.35)	0.13×10^6^ (38335.00–0.10×10^7^)	2338.54 (2338.42–2338.65)	1.78
	F21	4	1.86±0.23	1034.05 (664.63–1462.17)	18271.00 (8876.20–76320.00)	2526.35 (2526.20–2526.49)	4.35
	F22	4	2.04±0.23	1290.22 (1017.12–1601.71)	17848.00 (11037.00–37045.00)	3150.06 (3149.90–3150.21)	1.56

All of the concentration-mortality curves followed the probit model based on the χ^2^ goodness-of-fit test (*P*>0.05).

**Table 2:** Heritability estimate (*h^2^*) of spinosad resistance for a seven-generation spinosad-selected strain of the tomato borer *Tuta absoluta*.

Parameters	Generations F1–F7	
Response estimate	F_1_ CL_50_ (log) (µg a.i./mL)	0.41 (−0.39)
	F_7_ CL_50_ (log) (µg a.i./mL)	2200.00 (3.34)
	Response to selection (R)	0.53
Estimate of differential selection	Selection-surviving Individuals (*p*; %)	31.25
	Intensity of selection (i)	1.12
	Initial slope	1.85
	Final slope	1.16
	Phenotypic standard deviation (sF)	0.66
	Differential selection (S)	0.75
	Generations for 10-fold increase in resistance (G)	1.88
	Herdability (*h* ^2^)	0.71

### Stability of spinosad resistance

Although the selection for spinosad resistance was rapid, it reached a plateau after the 7^th^ generation of selection with no further increases in the level of resistance with additional selection maintained until the 22^nd^ generation (Fig. 1). After reaching a plateau in the selection for spinosad resistance, the selection was interrupted in a line of selected insects exhibiting high spinosad resistance (>100,000-fold based on standard-susceptible strain) and such high resistance levels quickly eroded with a negative rate of change in subsequent generations without selection (RC = −1.06). Spinosad resistance was, therefore, unstable without spinosad exposure and resulted in a 10-fold reduction in the level of spinosad resistance at each 1.57 generations with a return to susceptibility resembling the original strains after eight generations without selection (Fig. 1).

### Inheritance of spinosad resistance

**Figure 2:**
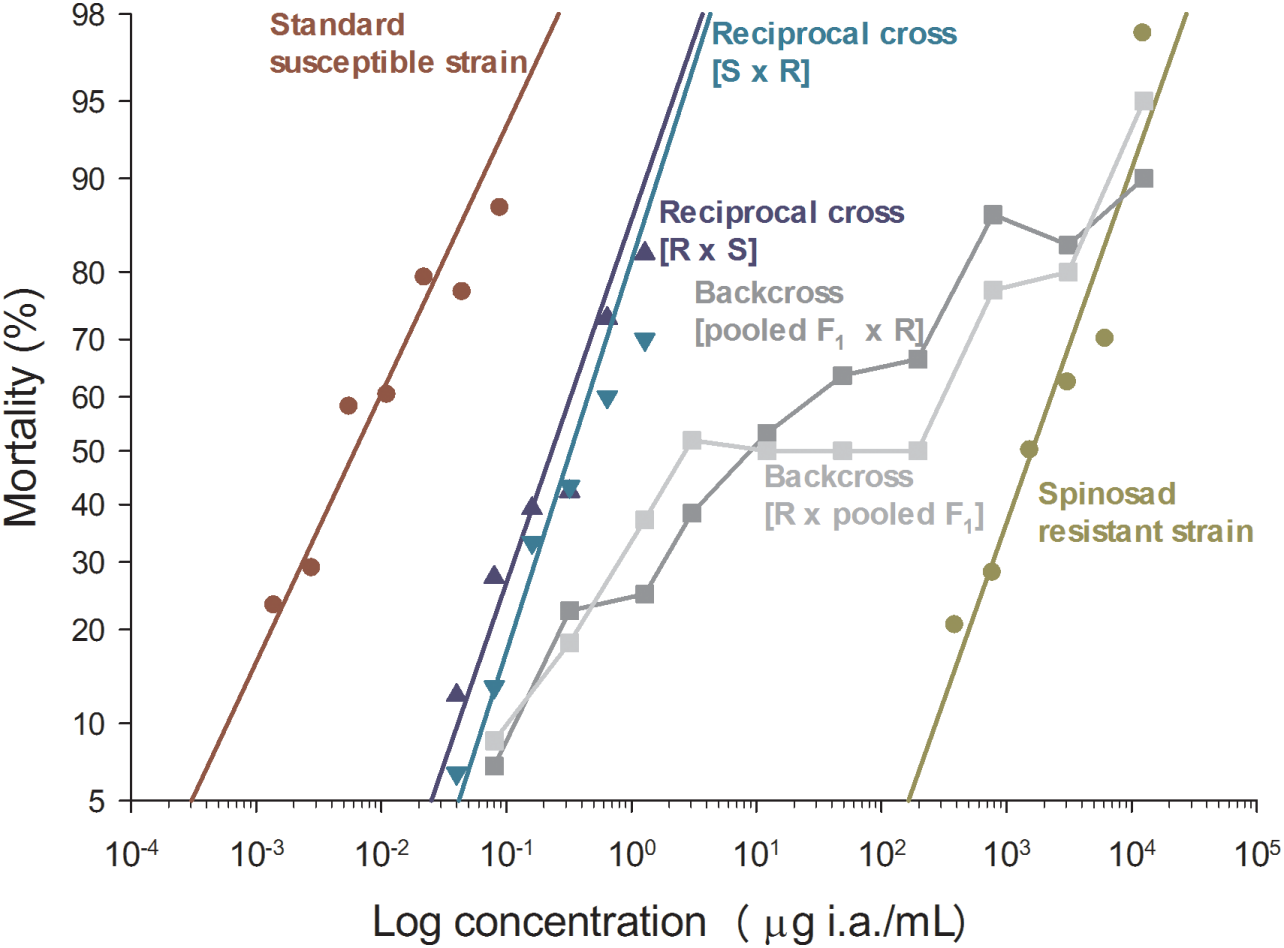
Spinosad concentration-mortality curves (with observed data as symbols) for the (standard) spinosad susceptible strain, (selected) spinosad resistant strain, the F_1_ progeny of the reciprocal crosses and the backcross progeny (pooled F_1_ RC×spinosad-resistant) of the tomato borer *Tuta absoluta*.

The spinosad-selected strain exhibited very high levels of spinosad resistance (180,000-fold) compared with the standard susceptible strain, both of which exhibited similar variability of responses based on the overlapping standard errors of the slope from the concentration-mortality curves of both strains, indicating their relatively similar homogeneity of responses to spinosad (i.e., they exhibit similar levels of homozygosis). The F_1_ progeny of the reciprocal crosses between spinosad-susceptible and –resistant strains exhibited intermediate levels of spinosad resistance (ca. 27- and 40-fold) (Table 3; Fig. 2), which were not significantly different based on the Polo-Plus χ^2^ test of equality of the concentration-mortality curves (χ^2^ = 3.53; df = 2, *P*>0.05). Therefore, spinosad resistance is an autosomal trait (i.e., not sex-linked) for the tomato borer.

**Table 3:** Relative toxicity of spinosad in spinosad-susceptible and (selected) spinosad-resistant strains, the progeny of reciprocal crosses (F_1_: ♀R×♂S and ♀S×♂R) and of backcrosses [F_1_ (pooled)×(selected) spinosad-resistant strain] of the tomato borer *Tuta absoluta*.

	No. insectstested		LC_50_ (95% CI)		Degree ofdominance (D)	LC_90_ (95% CI)		Degree ofdominance (D)	
Strain	(degrees offreedom)	Slope ± SE	(µg a.i./mL)	RR_50_ (95% CI)	at LC_50_ (±SE)	(µg a.i./mL)	RR_90_ (95% CI)	at LC_90_ (±SE)	?^2^
Spinosad susceptible	320	1.18±0.13	0.01	1.00	-	0.113	1.00	-	5.32
(standard)	(6)		(0.007–0.01)	(0.81–1.19)		(0.071–0.222)	(0.81–1.19)		
Spinosad-resistant (F_15_ of	238	1.47±0.19	1717.30	183122.81	-	12730.00	1354308.97	-	5.46
selected strain)	(4)		(998.00–2764.00)	(183123.17–182847.10)		(6481.00–56151.00)	(1354309.17–1354309.25)		
Reciprocal cross ♂ S×♀ R	216	1.51±0.19	0.26	27.27	−0.45±0.03	1.81	15.96	−0.52±0.06	5.32
	(5)		(0.17–0.39)	(27.65–27.44)		(1.02–4.98)	(27.65–27.44)		
Reciprocal cross ♂ R×♀ S	211	1.60±0.19	0.38	40.31	−0.39±0.03	2.38	21.03	−0.47±0.06	5.83
	(5)		(0.25–0.59)	(40.12–40.29)		(1.30–7.16)	(20.68–21.37)		
Pooled F_1_ of reciprocal	427	1.55±0.13	0.31	33.15	−0.42±0.02	2.11	18.67	−0.49±0.05	9.18
crosses (RC)	(5)		(0.21–0.46)	(33.48–33.25)		(1.22–5.32)	(18.34–18.94)		
Backcross (Pooled F_1_ RC×	347	0.57±0.05	18.15	1947.35	-	4314.1	38374.52	-	7.21
Spinosad-resistant)	(9)		(9.40–35.36)	(1947.03–1947.66)		(1479.10–18945.00)	(38373.96–38375.12)		

All of the concentration-mortality curves followed the probit model based on the χ^2^ goodness-of-fit test (*P*>0.05).

The degree of dominance of spinosad resistance was estimated for the F_1_ progeny of both reciprocal crosses between spinosad-susceptible and –resistant strains, and also for the pooled data from both progenies, providing values ranging from –0.39 to –0.45 (at the LC_50_). The estimates of degree of dominance at the LC_90_ for the same progenies were similar ranging from –0.47 and –0.52. These findings indicate that spinosad resistance is incompletely recessive, which was further confirmed by estimating the dominance using a range of five concentrations against the spinosad-susceptible and -resistant strains and their F_1_ progeny (pooled together from both reciprocal crosses strain) (Table 4). At high concentrations, full recessiveness prevailed, while at low concentrations full dominance prevailed and the incompletely recessive pattern prevailed at intermediate spinosad concentrations as would be expected for an incompletely recessive pattern of inheritance.

**Table 4:** Dominance of spinosad resistance based on a range of spinosad concentrations including LC_50_s from the susceptible parental strains and pooled F_1_ progeny of reciprocal crosses estimated for the tomato borer *Tuta absoluta*.

Concentrations (µg a.i./mL)	Strains	No. insects	Mortality (%)	Survival performance	Estimated dominance (*h*)
0.005	Spinosad-resistant	19	0.00	1.00	-
	Spinosad-susceptible	29	3.45	0.97	-
	Pooled F_1_ of reciprocal crosses	30	0.00	1.00	1.00
0.05	Spinosad-resistant	25	0.00	1.00	-
	Spinosad-susceptible	28	25.00	0.75	-
	Pooled F_1_ of reciprocal crosses	29	0.00	1.00	1.00
0.50	Spinosad-resistant	22	0.00	1.00	-
	Spinosad-susceptible	30	100.00	0.00	-
	Pooled F_1_ of reciprocal crosses	31	58.06	0.42	0.42
5.00	Spinosad-resistant	21	0.00	1.00	-
	Spinosad-susceptible	29	100.00	0.00	-
	Pooled F_1_ of reciprocal crosses	30	100.00	0.00	0.00
10.00	Spinosad-resistant	29	13.79	0.86	-
	Spinosad-susceptible	31	100.00	0.00	-
	Pooled F_1_ of reciprocal crosses	30	100.00	0.00	0.00

The concentration range used also discriminates for high spinosad resistance, as observed in the spinosad-selected strain. The estimated dominance (*h*) varies from 0 (completely recessive) to 1 (completely dominant), where 0.5 corresponds to co-dominance, 0<*h*<0.5 corresponds to incompletely recessive and 0.5<*h*<1.0 corresponds to incompletely dominant.

The direct test for monogenic inheritance of spinosad resistance provided non-significant variation between expected and observed frequencies at increasing spinosad concentrations (Table 5). The overall χ^2^ test for the 11 spinosad concentrations tested was not significant (χ^2^ = 14.88, df = 10, *P*>0.05) (Table 5). The minimal number of effective genes (n*_E_*) involved in spinosad resistance was 0.63 indicating, again, a monogenic trait.

**Table 5:** Direct test of monogenic inheritance for spinosad resistance in the tomato borer *Tuta absoluta* by comparing expected and observed mortality of the progeny of the backcrosses between the pooled F1 progeny of the reciprocal crosses and the (selected) spinosad-resistant strain.

Concentration (mg a.i./L)	Observed mortality (%)	Expected mortality (%)	?^2^ *	*P*
0.04	0.00	0.00	0.00	1.00
0.08	6.90	8.62	0.11	0.74
0.32	22.58	18.33	0.37	0.54
1.28	25.00	37.27	2.06	0.15
3.05	38.46	52.00	2.86	0.09
12.21	53.33	50.00	0.13	0.72
48.83	63.89	50.00	2.78	0.10
195.31	66.67	50.00	3.33	0.07
781.25	86.67	77.50	1.45	0.23
3125.00	83.33	80.00	0.21	0.65
12500.00	90.00	95.00	1.58	0.21
Total			Σχ^2^ = 14.89	0.14

*Non-significant at *P*>0.05.

### Spinosad cross-resistance spectrum

The concentration-mortality curves for nine different insecticides (of different groups) used against the tomato borer were estimated for the parental spinosad-resistant strain (Iraquara) and its spinosad-selected strain after 15 generations of selection to allow the recognition of potential patterns of cross-resistance (i.e., a single resistance mechanism leading to resistance to two or more insecticides). An eventual increase in resistance with the increase of spinosad resistance by selection indicates cross-resistance. However, among the insecticides tested, only spinetoram exhibited a significant increase in resistance with selection for spinosad resistance (Table 6). Therefore, cross-resistance was observed only between spinosad and spinetoram, another spinosyn insecticide.

**Table 6:** Relative toxicity of insecticides to the parental spinosad-resistant strain and its derived strain after 15-generations of selection for spinosad resistance.

Insecticides	No. insects	Slope ± SE	LC_50_ (95% CI) (µg a.i./mL)	RR_50_ (95% CI)	?^2^ (degrees of freedom)
**Parental spinosad-resistant strain**					
Spinosad	264	1.85±0.23	0.410 (0.31–0.51)	-	1.51 (5)
Spinetoram	276	1.72±0.18	0.29 (0.23–0.38)	-	2.91 (5)
Abamectin	282	1.56±0.24	0.54 (0.31–0.78)	-	2.07 (5)
Chlorantraniliprole	243	2.84±0.38	12.18 (9.38–15.10)	-	2.40 (4)
Cartap	265	2.25±0.25	173.65 (137.26–214.16)	-	0.95 (5)
Chlorfenapyr	283	1.65±0.21	1.08 (0.74–1.44)	-	0.77 (5)
Indoxacarb	279	3.25±0.47	0.86 (0.69–1.04)	-	0.41 (5)
Thiamethoxam	300	1.65±0.17	1008.86 (717.56–1389.27)	-	5.49 (5)
Permethrin	281	1.87±0.21	269.15 (204.91–342.36)	-	5.49 (6)
Chlorpyrifos	273	2.30±0.23	509.16 (416.99–623.34)	-	2.30 (5)
**Selected spinosad-resistant strain**					
Spinosad	238	1.47±0.19	1717.30 (998.16–2764.20)	4191.55 (4191.38–4191.72)	5.46 (4)
Spinetoram	211	1.62±0.23	195.94 (140.94–261.88)	656.99 (656.82–657.15)	2.32 (4)
Abamectin	282	1.66±0.18	2.85 (2.12–3.66)	5.25 (5.00–5.50)	2.15 (6)
Chlorantraniliprole	244	1.80±0.22	0.42 (0.30–0.55)	0.03 (0.01–0.20)	1.26 (5)
Cartap	243	1.21±0.20	105.34 (72.22–164.21)	0.61 (0.41–0.80)	0.32 (4)
Chlorfenapyr	432	1.19±0.12	3.80 (2.94–4.89)	3.53 (3.35–3.71)	3.86 (5)
Indoxacarb	313	1.68±0.19	1.19 (0.73–1.72)	1.38 (1.22–1.54)	6.64 (6)
Thiamethoxam	212	1.91±0.23	3573.35 (2414.90–5147.74)	3.54 (3.40–3.69)	4.40 (4)
Permethrin	238	1.62±0.19	662.07 (497.68–864.87)	2.46 (2.30–2.62)	2.12 (5)
Chlorpyrifos	299	1.82±0.19	951.97 (758.56–1221.65)	1.87 (1.73–2.00)	4.75 (5)

All of the concentration-mortality curves followed the probit model based on the χ^2^ goodness-of-fit test (*P*>0.05).

### Synergism of spinosad

**Figure 3:**
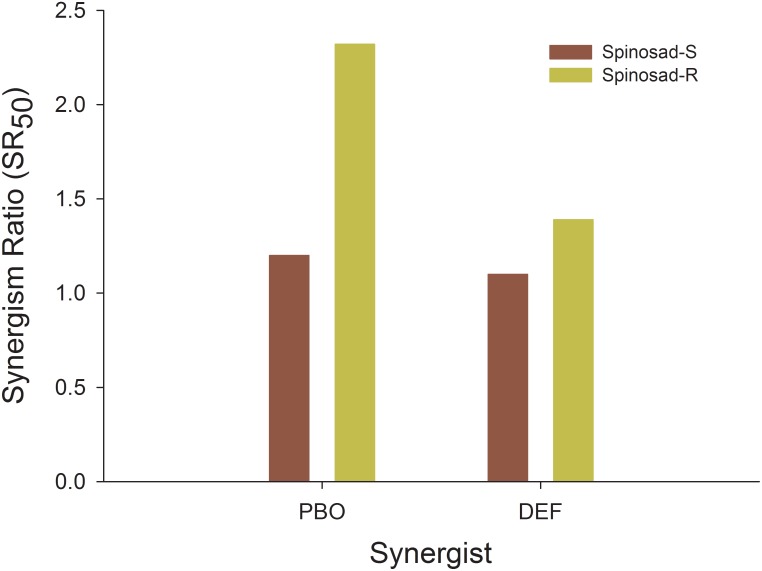
Synergism of spinosad toxicity in spinosad-susceptible and -resistant strains of the tomato borer *Tuta absoluta*.

The synergism ratios with PBO and DEF were 1.2- and 1.1-fold, respectively (Fig. 3) for susceptible colony, while it was 2.32- and 1.39-fold in the resistant colony for PBO and DEF, respectively. Therefore, the synergisms caused by PBO and DEF against spinosad for the resistant colony were 1.93- and 1.26-fold greater compared to the susceptible colony, respectively. This suggests that such enzymes play a minimal role in the spinosad resistance.

### Activity of detoxification enzymes

**Figure 4:**
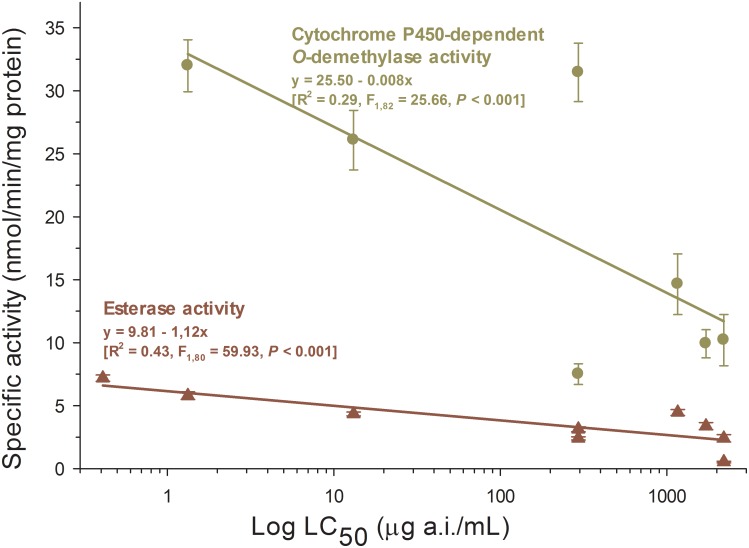
Relationship between detoxification enzyme activity and LC_50_s for spinosad in spinosad-selected generations of the tomato borer *Tuta absoluta*.

The activity of esterases and cytochrome P450-dependent monooxygenases was determined for the spinosad-selected strain after different generations of selection to identify a potential increase in detoxification activity with the increase in spinosad resistance. However, the activity of both detoxification enzymes significantly decreased with selection for spinosad resistance indicating that they are not the underlying mechanism of this phenomenon (Fig. 4).

## Discussion

In countries managing invasive species and maintaining records of insecticide resistance development, insecticide use against the tomato borer can be described as waves of use of different (insecticide) groups. This is the case in Chile and Brazil, where the initial use of organophosphates, pyrethroids, and cartap was replaced by abamectin and subsequently by insect growth regulators as the main insecticide groups under use [10], [12]–[16], [19]–[21]. The main determinant in the replacement of an insecticide is the sequential development of insecticide resistance to the main insecticides being utilized at a given time, this phenomenon has evolved quickly in the tomato borer leading to control failures and relatively fast changes in the patterns of insecticide use [10], [12]–[17], [19]–[21]. Three insecticides are the main compounds currently being used against the tomato borer in Brazil, the spinosyn spinosad, the pyrrole chlorfenapyr, and the diamide chlorantraniliprole [18]–[21].

Quick reselection for resistance to insecticides has limited the range of available compounds for managing the tomato borer, increasing reliance on few molecules for this objective [20], . The concern of spreading insecticide resistant phenotypes of the tomato borer justifies the examination of the occurrence of insecticide resistance in this species. The new focus is on the few compounds under effective use, particularly in the likely centers of spread of the species. The emergence of spinosad resistance in South America is cause for concern, and the levels of resistance seemed to have increased quickly, but there is little information available beyond an initial survey [19]–[23]. The quick development of spinosad resistance in the region is suggestive of a highly inheritable (monogenic) trait, which was confirmed in our study.

Very high levels of spinosad resistance (>180,000-fold) were achieved within seven generations of selection from a field population already exhibiting resistance to spinosad [23]. The heritability of spinosad resistance proved high, with enough field variability to allow a quick selection for resistance, which has also been observed for spinosad resistance in the diamondback moth *Plutella xylostella* (L.) and the American serpentine leafminer *Liriomyza trifolii* (Burgess) [45], [46]. A monogenic pattern of inheritance is consistent with the fast selection and evolution of spinosad resistance and such autosomal monogenic inheritance was observed in the tomato borer, which was incompletely recessive. This inheritance seems to be the general pattern for spinosad resistance in insect pest species [47]–[49]. The simple inheritance and high heritability of spinosad resistance in the tomato borer reinforces the phytosanitary concerns of the quick dispersion of this pest species and the spread of insecticide resistant phenotypes or populations amenable to fast local selection for spinosad resistance [19], [31].

The potential cross-resistance to other insecticidal compounds is another issue of concern because it may further limit the management tools available against a pest species, particularly an invasive and very destructive species that is already difficult to control, such as the tomato borer [8]–[10], [19]. Cross-resistance in spinosad-resistant insect populations seems limited to related compounds [50], [51], as observed for the tomato borer where only cross-resistance to spinetoram, another spinosyn, was observed. This pattern of cross-resistance among spinosyns has been associated with altered target site sensitivity in the insect strains resistant to these compounds and their site of action [50]. This is consistent with our findings in the tomato borer, despite an earlier suggestion of the potential involvement of enhanced cytochrome P450-dependent monooxygenase activity in spinosad resistance in this species [22]. Initial correlational evidence of the potential involvement of enhanced esterase activity in spinosad resistance also suggested enhanced detoxification as a potential mechanism [23]. However, these earlier suggestions did not provide substantiated evidence for this possibility and our results did not support this hypothesis. Our results demonstrated altered target site sensitivity as the underlying mechanism of spinosad (and spinetoram) resistance in the tomato borer [50], [52].

Spinosad resistance evolved quickly in the tomato borer under spinosad pressure reaching a high threshold of selection with over a level of 180,000-fold level of resistance, maintained with continued selection. However, the interruption of spinosad selection led to a quick erosion of spinosad resistance reestablishing the initial (reduced) levels of resistance after eight generations. This finding indicates that there is a fitness cost associated with insecticide resistance, which has been reported in different insect species and with different insecticides [46], [53]–[55]. The fitness disadvantage of spinosad resistant populations of the tomato borer without selection pressure by spinosyn applications allows for the potential of moderation as an insecticide resistance management strategy. In this case, the reduction of spinosad use for a few generations of the tomato borer (>10) will allow the eventual reestablishment of susceptibility to spinosad and the subsequent reuse of this insecticide in the area. Reselection for spinosad resistance is likely to be rapid based on what has been observed for other insecticides in the tomato borer and in other species [4]–[7], [20], [21], but insecticide rotation with compounds of different modes of action and detoxification should extend the field use of spinosyns against the tomato borer.

In summary, very high levels of spinosad resistance were quickly selected for in the tomato borer with a monogenic autosomal pattern of inheritance that was incompletely recessive. A cross-resistance spectrum to spinetoram, another spinosyn, was observed, which suggested that the likely resistance mechanism involved is an altered target site sensitivity, given that the activity of esterases and cytochrome P450-dependent monooxygenases were not associated with spinosad resistance in the tomato borer. Spinosad resistance was unstable without spinosad selection, suggesting that the suspension of spinosyn use against the tomato borer would be a useful component in spinosad resistance management for this species.

Spinosad use against this species in introduced areas should be carefully monitored to prevent rapid selection for high levels of resistance and the potential for its spread to new areas.
